# Influence of Salt Stress on Growth of Spermosphere Bacterial Communities in Different Peanut (*Arachis hypogaea* L.) Cultivars

**DOI:** 10.3390/ijms21062131

**Published:** 2020-03-20

**Authors:** Yang Xu, Dai Zhang, Liangxiang Dai, Hong Ding, Dunwei Ci, Feifei Qin, Guanchu Zhang, Zhimeng Zhang

**Affiliations:** 1 Shandong Peanut Research Institute, Shandong Academy of Agricultural Sciences, Qingdao 266100, China; xy52120092661@163.com (Y.X.);; 2 College of Plant Protection, Hebei Agricultural University, Baoding 071001, China; adaiadai.1987@163.com

**Keywords:** bacterial community diversity, peanut (*Arachis hypogaea* L.), peanut cultivars, salt stress, spermosphere

## Abstract

Background: Exposure of seeds to high salinity can cause reduced germination and poor seedling establishment. Improving the salt tolerance of peanut (*Arachis hypogaea* L.) seeds during germination is an important breeding goal of the peanut industry. Bacterial communities in the spermosphere soils may be of special importance to seed germination under salt stress, whereas extant results in oilseed crop peanut are scarce. Methods: Here, bacterial communities colonizing peanut seeds with salt stress were characterized using 16S rRNA gene sequencing. Results: Peanut spermosphere was composed of four dominant genera: *Bacillus*, *Massilia*, *Pseudarthrobacter*, and *Sphingomonas*. Comparisons of bacterial community structure revealed that the beneficial bacteria (*Bacillus*), which can produce specific phosphatases to sequentially mineralize organic phosphorus into inorganic phosphorus, occurred in relatively higher abundance in salt-treated spermosphere soils. Further soil enzyme activity assays showed that phosphatase activity increased in salt-treated spermosphere soils, which may be associated with the shift of *Bacillus*. Conclusion: This study will form the foundation for future improvement of salt tolerance of peanuts at the seed germination stage via modification of the soil microbes.

## 1. Introduction

Peanut or groundnut (*Arachis hypogaea* L.), an important nutritious food and cash crop, is consumed both as oilseed and livestock fodder, forming an important revenue source for farmers [1,2]. As a moderately salt-sensitive species, cultivation of peanuts was attempted in low-salinity fields in China to gain more crop production [3,4]. Peanuts growing in these types of fields are impaired by soil salinity throughout their life cycle and their seed germination is the more sensitive stage to salt stress [3,5]. Soil salinity can create osmotic potential around the seed and cause excessive ion toxicity (Na^+^ and Cl^−^) to seeds [6,7,8]. Therefore, the exposure of seeds to soil salinity results in lower seed viability, reduced germination percentage, increased germination time, and even total inhibition of germination [5,9]. Seed germination is not only highly related to the seedlings’ survival rate but also subsequent vegetative growth. Therefore, enhancing salt tolerance at the seed germination stage has become a major breeding goal in the current agricultural industry.

Germination starts with the uptake of water by the dry seed and ends with radicle emergence, which is an initial stage in the plant’s life cycle [10,11]. After being dispersed from the mother plant, seed undergoes continuous desiccation in nature, and germination is triggered upon meeting proper environmental conditions [11]. A previous study showed that germination initiation depended on the regulation of plant phytohormones, such as gibberellic acid (GA), abscisic acid (ABA), ethylene, and auxin [12,13,14,15]. Moreover, some genes related to GA and ABA biosynthesis and signaling also altered seed dormancy and germination [12,16]. Knowledge of the in-depth molecular mechanism that integrates seed germination with salt stress is scarce.

During imbibition and germination, nutrients are released primarily from the embryo end of the seed closest to the emerging radicle, and these nutrients can attract a great number of microbes, allowing a bacterial community to be established on seeds [17]. The specific zone surrounding seeds is named as the spermosphere, where interactions between the soil, seed-borne bacterial communities, and germinated seeds take place [18,19,20]. Various studies have emerged that implicate that spermosphere bacterial communities are involved in regulating seed germination [19,21,22,23]. However, the potential role of seed-associated bacteria on seed germination under salt stress are poorly characterized. Some beneficial microbes in the rhizosphere bacterial community possess diverse metabolic capabilities and play crucial roles in plant adaptation to environmental stresses [24,25,26,27]. Thus, we hypothesize that spermosphere bacterial communities may also have implications for seed germination and survival under salt stress, which requires in-depth study.

To detect and provide new insight into the influence of salt stress and different peanut cultivars on the composition of the peanut spermosphere bacterial community, 16S rRNA gene sequencing and integrated taxonomic data were performed in this study. The study aimed to identify the most favorable stress-associated spermosphere bacterial species and further illustrate their potential roles in empowering seeds to overcome salt stress. Therefore, inoculating seeds with high doses of specific beneficial microbes may be a good strategy to activate seed germination and potentially empower the seeds to overcome environmental stress in future.

## 2. Results

### 2.1. Overall and Alpha Diversity Analysis of 16S rRNA Gene Sequencing Data

**Figure 1:**
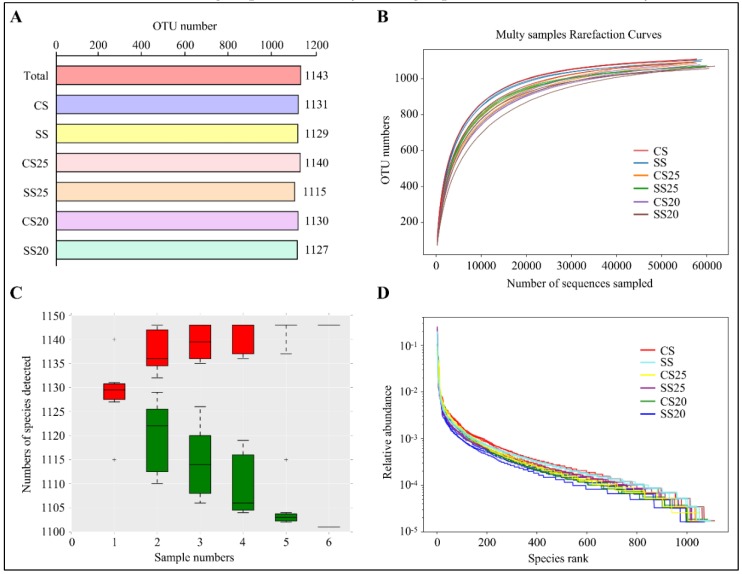
Overall sequence data and alpha diversity analysis. (**A**) Operational taxonomic units (OTUs) of different soil groups. CS, control bulk soil; SS, salt-treated bulk soil; CS25, controlled spermosphere soil around Huayu25 (salt-resistant peanut cultivar); CS20, controlled spermosphere soil around Huayu20 (salt-susceptible peanut cultivar); SS25, salt-treated spermosphere soil around Huayu25; SS20, salt-treated spermosphere soil around Huayu20. (**B**) Rarefaction curve analysis showing the gene sequencing depth. (**C**) Species accumulation curves showing the rate of increase of new species with the increase in sample size. “+” represents extreme outliers. Single red box reflects the total number of species in the sample, and all the red boxes form the accumulation curve. The single green box reflects the number of common species in the sample, and all the green boxes form the common quantity curve. (**D**) Rank abundance curve showing the relative species abundance and evenness. The length of the polyline on the horizontal axis reflects the OTU numbers and represents the richness of the bacterial community. The flatness of the polyline reflects the evenness of the bacterial community composition.

To explore the influence of salt stress and different peanut cultivars on the composition of the spermosphere bacterial community, we sequenced the bacterial genomes around various peanut seeds under normal and salt stress conditions. We defined the salt-treated spermosphere soil around Huayu25 (salt-resistant peanut cultivar) and Huayu20 (salt-susceptible peanut cultivar) as salt-treated spermosphere 25 (SS25) and SS20, respectively. Additionally, controlled spermosphere soil around Huayu25 and Huayu20 were defined as CS25 and CS20, respectively. Moreover, salt-treated (SS) and control (CS) bulk soil without plants were also collected as the negative control. After removing any doubtful sequences, 1,243,849 sequences passed quality screening and most of the sequence lengths were found to be between 390 and 450 bp (Figure S1). Then, the high-quality reads were clustered in a total of 1143 different operational taxonomic units (OTUs) with a taxonomic assignment evaluated with >97% sequence identity as the cut-off. The complete lists of OTUs per soil group are shown in Figure 1A, where SS25 had the lowest OTU numbers and CS25 contained the most.

Species richness and evenness within a single bacterial ecosystem of the peanut spermosphere were examined via alpha diversity analysis. The rarefaction curve was the common method to examine species diversity in the ecosystem. Results found that each sample of the peanut spermosphere exhibited highly diversified bacterial communities on the basis of the fact that the rarefaction curves of these samples did not approach the asymptote (Figure 1B). The species accumulation curves can reflect the sequencing depth. As shown in Figure 1C, the increase rate of new species followed with the increase in sample size during the sampling process, implying that the sequencing depth was high enough to observe community richness. Moreover, rank abundance curves showed that all the soil groups had high species evenness and homogeneity (Figure 1D). These data indicate that all the soil groups in our study had high species richness and diversity.

### 2.2. Differences in the Peanut Spermosphere Bacterial Community Structure

**Figure 2:**
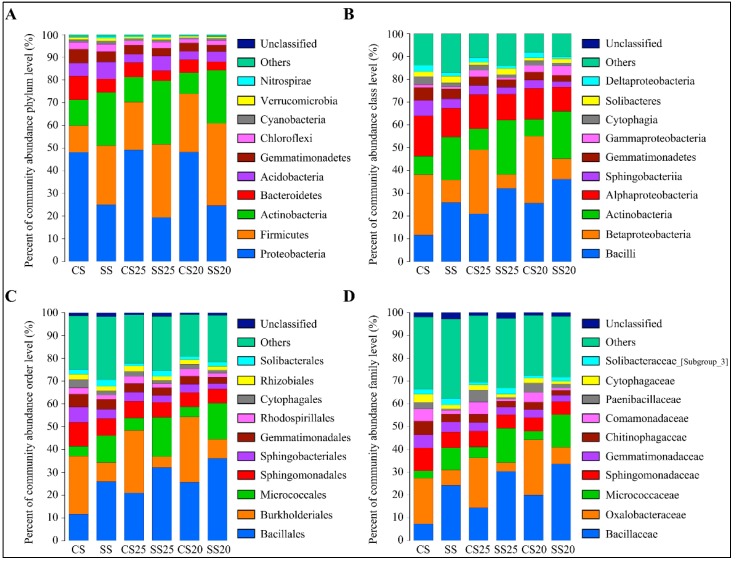
Bacterial community structure in the peanut spermosphere soils and the bulk soils at the phylum, class, order, and family level. (**A**) Percent of taxa at the phylum level in the peanut spermosphere soils and the bulk soils. The relative abundance was calculated by averaging the abundances of three duplicates in each soil group. (**B**) Percent of taxa at the class level in the peanut spermosphere soils and the bulk soils. The relative abundance was calculated by averaging the abundances of three duplicates in each soil group. (**C**) Percent of taxa at the order level in the peanut spermosphere soils and the bulk soils. The relative abundance was calculated by averaging the abundances of three duplicates in each soil group. (**D**) Percent of taxa at the family level in the peanut spermosphere soils and the bulk soils. The relative abundance was calculated by averaging the abundances of three duplicates in each soil group.

To further analyze the bacterial community structure, the specific composition of each sample at five levels of classification (phylum, class, order, family, and genus) was developed. The high-quality classifiable sequences were affiliated with 10 bacterial phyla using the phylogenetic assignment tool (Figure 2A). Although the abundance of phyla varied in the peanut spermosphere soil samples around different peanut cultivars with or without salt treatment, Proteobacteria, Firmicutes, Actinobacteria, and Bacteroidetes were the most abundant bacteria in all the soil groups, accounting for over 80% of the bacterial taxa (Figure 2A). Firmicutes increased in the soils around peanut seeds compared to the bulk soil without plants (Figure 2A). Salt stress can alter peanut spermosphere soil bacterial community structure. The presence of Firmicutes and Actinobacteria gradually increased in SS25 and SS20 as predominant bacteria, whereas quantities of Proteobacteria and Bacteroidetes decreased in these soils compared with CS25 and CS20 (Figure 2A). At the class level, Bacilli, Betaproteobacteria, Actinobacteria, Alphaproteobacteria, and Sphingobacteriia were the five classes that predominated in all the soil groups. Bacilli and Actinobacteria were the dominant classes in SS25 and SS20, whereas Betaproteobacteria and Alphaproteobacteria decreased in these salt-treated spermosphere soils (Figure 2B). The relative percentage abundance analyses revealed that the most dominant order was Bacillales then Burkholderiales, followed by Micrococcales and Sphingomonadales (Figure 2C), and the abundance and identity of the reads suggested that strains taxonomically related to Bacillaceae, Oxalobacteraceae, Micrococcaceae, and Sphingomonadaceae at the family level were present in the peanut spermosphere soils and the bulk soils in high abundance (Figure 2D).

**Figure 3:**
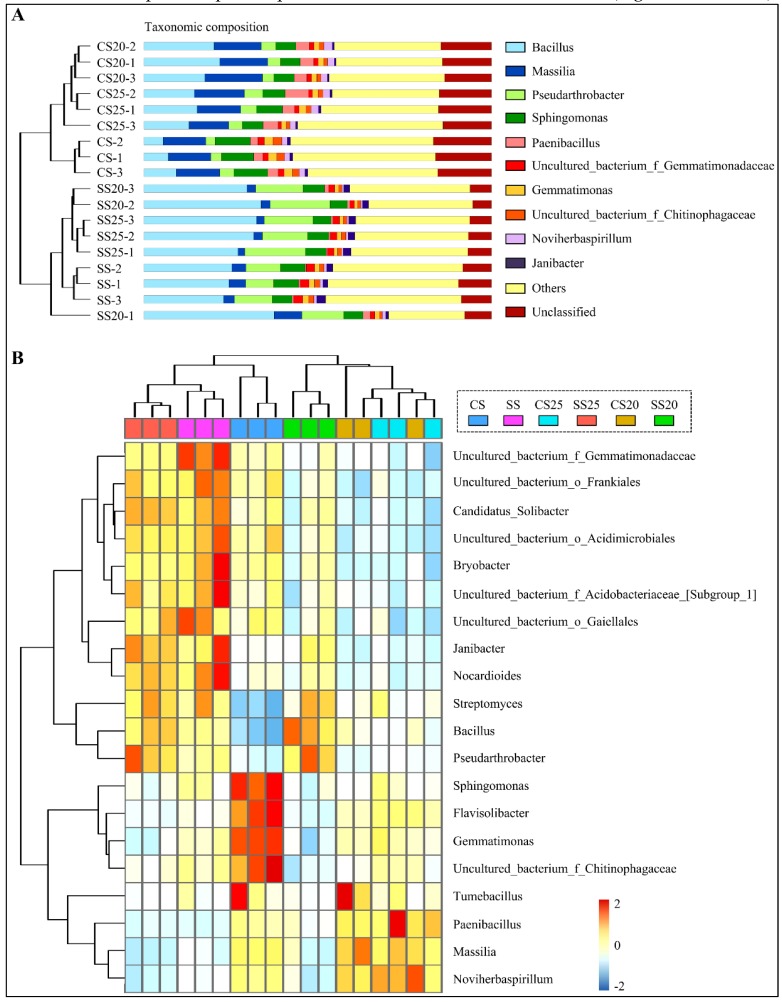
Bacterial community diversity analysis through bar charts and heatmap. (**A**) Percent of taxa at the genus level in peanut spermosphere soils and the bulk soils is visualized using bar charts. Samples are clustered according to the similarity among their constituents and arranged in vertical order. The shorter the branch length between samples, the higher the similarity of the two samples. (**B**) The relative abundance of the top 20 abundant genera is visualized using a heatmap. Samples are clustered according to the similarity among their constituents and arranged in a horizontal order. The taxa are also clustered according to the degree of similarity distributed among different soil samples and arranged in a vertical order. The red rectangle represents the more abundant genera and the blue rectangle represents the less abundant genera.

A thorough investigation at the genus level was also performed, and the top 10 most abundant genera are shown in Figure 3A and Figure S2. *Bacillus*, *Massilia*, *Pseudarthrobacter*, *Sphingomonas*, and *Paenibacillus* were predominantly found in all the soil groups. Furthermore, many unclassified sequences were also abundant in these soil samples (6.39–16.08%) (Figure 3A and Figure S2), demonstrating that the soil remains a challenging reservoir of bacterial diversity. Clustering analysis showed that genus abundance varied from various soil groups, and the salt-treated samples were farther apart from the untreated soil samples (Figure 3A and Figure S2). Further structure analysis showed that bacterial community abundance indeed altered markedly with salt stress, whereas the differences between the two peanut cultivars were relatively small. The abundance of *Bacillus* and *Pseudarthrobacter* significantly increased in SS25 and SS20, rising by 68.72–110.72% and 282.14–219.21% compared with that of CS25 and CS20, respectively (Table S1). In contrast, the number of genus *Massilia* was decreased in SS25 and SS20 compared to the CS25 and CS20 (Figure 3A and Figure S2). Interestingly, the top 10 dominant genera occupied a greater proportion in the soils around peanut seeds than in the bulk soils, showing an enrichment trend of dominant bacterial groups in the peanut spermosphere soils. Moreover, salt stress can also elevate the proportion of the top 10 predominant bacterial communities in the peanut spermosphere soils and bulk soils to some extent (Figure 3A and Figure S2).

Phylogenetic tree analysis showed that the top 20 most abundant genera belonged to the phyla of Actinobacteria (7/20), Firmicutes (3/20), Acidobacteria (3/20), and Proteobacteria (3/20) (the proportion of genera in specific phylum was shown in brackets) (Figure S3). A hierarchically clustered heatmap based on the abundance distribution of taxa or the degree of similarity among the six soil groups at the genus level was used to identify the different compositions of peanut spermosphere soils. Consistently, *Bacillus* and *Pseudarthrobacter* at the genus level were relatively abundant in SS25 and SS20 as compared to CS25 and CS20. *Flavisolibacter*, *Gemmatimonas*, *Sphingomonas*, and *uncultured_bacterium_f_Chitinophagaceae* were only particularly abundant in the bulk soil (Figure 3B). In a horizontal order, samples of CS20 and CS25 were tightly clustered according to the similarity among their constituents, whereas samples of SS20 and SS25 were farther apart. More precisely, the number of *Nocardioides*, *Janibacter*, *Bryobacter*, and some unclassified sequences were divergent between SS20 and SS25, suggesting that salt stress may widen the gap of bacterial community structure between two peanut cultivars. Altogether, these data suggest that peanut spermosphere bacteria may respond more dramatically to salt stress than peanut cultivars, leading to the shift of bacterial community composition.

### 2.3. Beta Diversity Analysis of Bacterial Community

**Figure 4:**
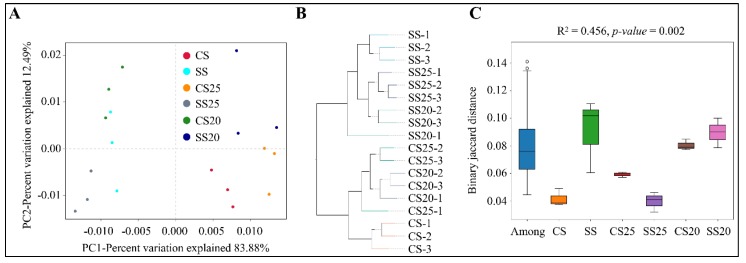
Beta diversity analysis. (**A**) Principal component analysis (PCA). Two principal components (PC1 and PC2) of PCA are shown in the coordinates. The same color points belonged to the same soil group are closer to each other and the samples from different soil groups are farther apart. (**B**) Unweighted pair-group method with arithmetic mean (UPGMA) analysis is clustered according to samples’ similarity. The longer the branch length between samples, the more variable the two samples are. (**C**) Analysis of similarities (ANOSIM) revealed the variation in the composition (Bray–Jaccard distance) of spermosphere or bulk soil bacterial communities.

In order to observe the similarities and dissimilarities among the soil samples, beta diversity analysis was also performed, including principal component analysis (PCA), unweighted pair-group method with arithmetic mean (UPGMA), and analysis of similarities (ANOSIM) analysis. PCA is a technique for analyzing and simplifying data by reflecting the differences among groups of data in two-dimensional coordinates. Distinct differences in bacterial communities were detected among six soil groups, and the first two principal components (PC1 and PC2) of PCA explained 83.88% and 12.49% of the total variation, respectively (Figure 4A). UPGMA analysis revealed that the bacterial community structures of various soil groups were diverse and the salt-treated samples were farther apart from the untreated samples, whereas three replicates in most of the soil groups (NS, SS, CS20, CS25, and SS25), and two duplicates of SS25 tightly clustered together (Figure 4B). Moreover, ANOSIM analysis was also performed, and the *R^2^* value was 0.452 and the *p*-value was 0.002 for binary jaccard distance (Figure 4C). Beta diversity analysis showed an obvious separation of the bacterial community composition under different treatments, suggesting that salt stress indeed alters the peanut spermosphere bacterial community structure.

### 2.4. Specific Phylotypes of Peanut Spermosphere Modulate by Salt Stress

**Figure 5:**
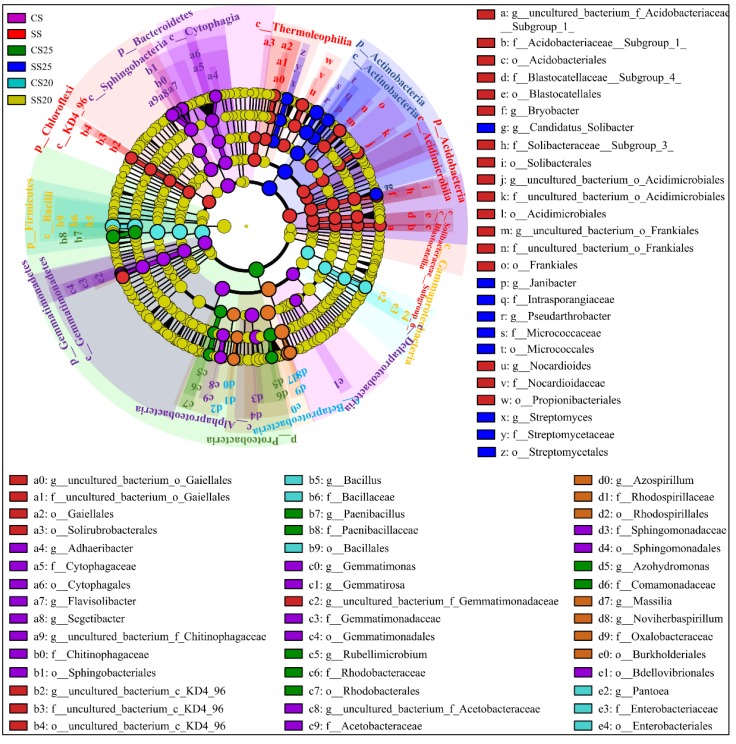
Cladogram showing specific phylotypes of bacterial community compositions of peanut spermosphere responding to salt stress. Circles indicate phylogenetic levels from phylum to genus (from the outer circle to the inner circle). The diameter of each circle is proportional to the abundance of the bacterial group.

Linear discriminant analysis (LDA) effect size (LEfSe) as an algorithm for high-dimensional biomarker discovery and explanation of specific phylotypes of peanut spermosphere responding to salt stress was explored in our study. Statistical analysis was performed from the phylum to the genus level in cladograms, and LDA scores of 3.5 or greater were confirmed by LEfSe (Figure 5 and Figure S4). In CS, Gemmatimonadates (from phylum to family, namely, p__Gemmatimonadetes, c__Gemmatimonadetes, o__Gemmatimonadales, and f__Gemmatimonadaceae), Sphingomonadales (from class to family, namely, c__Sphingobacteriia, o__Sphingobacteriales, and f__Sphingomonadaceae), Cytophagales (from class to family, that is, c__Cytophagia, o__Cytophagales, and f__Cytophagaceae), and two classes of Proteobacteria (c__Alphaproteobacteria and c__Deltaproteobacteria) were dominant bacteria. In SS, phyla Chloroflexi and Acidobacteria were evidently abundant (Figure 5). Moreover, the predominant bacterial community was diverse in the peanut spermosphere soils of different treatments. In untreated spermosphere soils, phyla Proteobacteria and Rhodobacterales (from order to genus, namely, o__Rhodobacterales, f__Rhodobacteraceae, and *g__Rubellimicrobium*) dominated in CS25, whereas Betaproteobacteria class specifically elevated in CS20 (Figure 5). After salt stress treatment, Actinobacteria (from phylum to genus, namely, p__Actinobacteria, c__Actinobacteria, o__Micrococcales, f__Micrococcaceae, and *g__Pseudarthrobacter*) dominated in SS25, and Firmicutes (from phylum to genus, namely, p__Firmicutes, c__Bacilli, o__Bacillales, f__Bacillaceae, and *g__Bacillus*) were predominant in SS20 (Figure 5). The bacterial community structure shows significant specificity under different treatments, which may be associated with the specific seed exudates and their survival abilities under salt stress.

### 2.5. Metabolic Functional Features of the Peanut Spermosphere Bacterial Community

**Figure 6:**
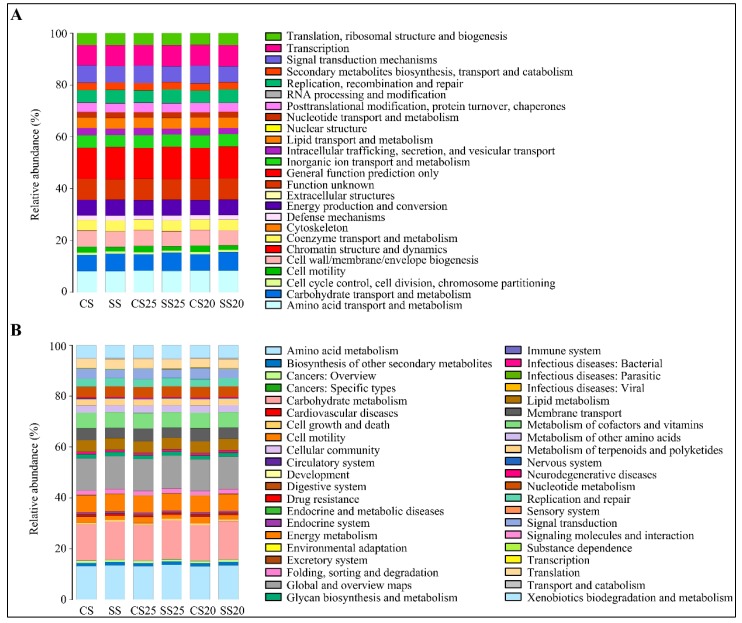
Metabolic functional features of the peanut spermosphere bacterial community. (**A**) Bar chart showing the relative abundance and diversity of functional groups in various peanut spermosphere soil groups and bulk soil groups in the context of the Cluster of Orthologous Groups (COG) database. Different COG groups are displayed in different colors, as listed in the right. (**B**) The Kyoto Encyclopedia of Genes and Genomes (KEGG) database showing the relative abundance and diversity of functional groups in various peanut spermosphere soil groups and bulk soil groups.

Metabolic functional features of the peanut spermosphere bacterial community were predicted via PICRUSt10 (phylogenetic investigation of communities by reconstruction of unobserved states) in the context of the Cluster of Orthologous Groups (COG) database. Stress response categories such as replication, recombination and repair, and defense mechanisms were predicted as being higher in SS25 and SS20 than that of CS25 and CS20 (Figure 6A and Table S2). These changing function groups may be closing with relation to salt stress.

The Kyoto Encyclopedia of Genes and Genomes (KEGG) database was also utilized to predict the function of bacterial population metabolism. Some metabolic functions (carbohydrate transport and metabolism; lipid metabolism and metabolism of terpenoids and polyketides) were enriched in the salt-treated soil groups (Figure 6B). Interestingly, the functional group (replication and repair) and defense-related group (xenobiotics biodegradation and metabolism) in the bacterial community of SS25 and SS20 were also significantly higher than that of CS25 and CS20, which is consistent with the COG analysis (Figure 6B and Table S3). These vigorous function groups of the bacterial community may be beneficial to peanut stress response at the seed germination stage.

### 2.6. The Response of Peanut Spermosphere Soil Extracellular Enzyme Activities to Salt Stress

**Figure 7:**
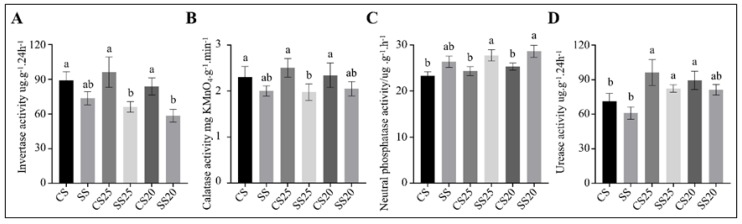
Soil enzyme activities of the peanut spermosphere soils and the bulk soils. Soil (**A**) invertase, (**B**) calatase, (**C**) neutral phosphatase, and (**D**) urease activities of the peanut spermosphere soils and the bulk soils. Error bars indicate the SEM (*n* = 3). One-way ANOVA Duncan’s test. Different lowercase letters represent different significance.

Extracellular soil enzyme activities are indicators of bacterial community health and soil quality [28]. To determine whether extracellular enzyme activities were altered with the significant changes in the functional potential of the soil bacterial communities, we measured the soil enzyme activities of various soil samples. Invertase and catalases are correlated with soil respiratory intensity and bacterial activity [29,30]. We found that catalase activity was roughly identical in all the soil groups, whereas the average invertase activity in SS25 and SS20 decreased by 35.5% and 28.6% compared with CS25 and CS20, respectively (Figure 7A,B). These findings suggest that bacterial life activities tend to be more vigorous to some extent in untreated spermosphere soils. Moreover, soil phosphatase activity and urease activity were also examined. We found that neutral phosphatase activity was slightly elevated in SS25 and SS20 compared with CS25 and CS20, whereas urease activity in four peanut spermosphere soils appeared higher than in the bulk soils (Figure 7C,D). Soil phosphatase can hydrolyze soil organic phosphate into inorganic phosphate for plants [31]. The results suggest that the higher soil phosphatase activity is conducive to improving soil phosphorus supply capacity and may be beneficial to alleviate salt stress during seed germination to some extent.

### 2.7. Quantitative PCR of Specific Bacterial Groups

**Figure 8:**
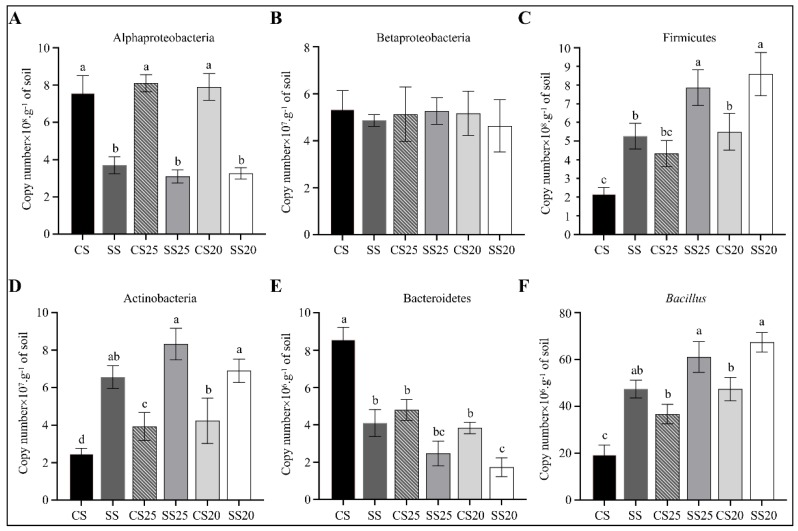
Quantification of predominant phyla or genera in the peanut spermosphere soils and the bulk soils. Quantification of (**A**) Alphaproteobacteria, (**B**) Betaproteobacteria, (**C**) Firmicutes, (**D**) Actinobacteria, (**E**) Bacteroidetes, and (**F**) *Bacillus* in various peanut spermosphere soils and bulk soils. Error bars indicate the SEM (*n* = 3). One-way ANOVA Duncan’s test. Different lowercase letters represent different significance.

To further verify the 16S rRNA gene sequencing data, we measured the changes in the abundance of the main bacterial phyla (Proteobacteria, Firmicutes, Actinobacteria, and Bacteroidetes) and the most predominant beneficial bacteria (*Bacillus*) in all the six soil groups using quantitative PCR (qPCR). Obvious enrichment of phylum (Firmicutes and Actinobacteria) and genus (*Bacillus*) were found in spermosphere soils and salt-treated bulk soils compared with that of CS. Moreover, the abundance of Firmicutes, Actinobacteria, and *Bacillus* were significantly higher in SS25 and SS20 compared with CS25 and CS20, whereas Proteobacteria and Bacteroidetes were lower in these salt-treated peanut spermosphere soils (Figure 8). The abundances of Betaproteobacteria were roughly identical in different soil groups. These results were consistent with most of the results of the 16S rRNA gene sequencing analysis.

## 3. Discussion

Numerous studies report the presence of seed microorganisms but few have examined the presence of these microbes around plant seeds, especially the oilseed crop peanut [32,33]. In this study, 16S rRNA gene sequencing was used to examine the bacterial communities of the spermosphere around various peanut cultivars with or without salt stress. The soil bacterial community structure of peanut consisted mainly of Proteobacteria, Firmicutes, Actinobacteria, and Bacteroidetes at the phylum level (Figure 2). Proteobacteria is the most abundant phylum in the peanut spermosphere, which may be due to their rapid growth rates [34]. In the previous study, Actinobacteria, Firmicutes, and Proteobacteria dominated in the spermosphere soil of spinach (*Spinacia oleracea*) [18]. Proteobacteria and Firmicutes were more abundant microbes than other microbes in the spermosphere soil of maize [17]. Proteobacteria, Actinobacteria, and Firmicutes were the mutually dominant bacterial phyla of peanuts and the above plants, indicating that they are the most common dominant bacterial phyla in plant spermosphere soils. In our recent data, Proteobacteria and Actinobacteria dominated in rhizosphere soil of peanut, which suggested that Proteobacteria and Actinobacteria are the two most abundant bacteria in the soil around peanut [1]. The biological function of the two phyla in peanut soils needs further research. However, Bacteroidetes was the specifically predominant phylum in the peanut spermosphere soils, which may be a result of specific seed exudates of peanuts.

In this study, bacterial community structure was markedly altered after salt stress treatment, whereas the differences between two peanut cultivars were relatively small (Figure 2 and Figure 3). The bacterial community constituents of CS25 and CS20 were similar, whereas the compositions of SS25 and SS20 were diverse (Figure 3B), which may be due to the different seed exudates from the salt-susceptible (Huayu20) and salt-resistant (Huayu25) cultivars under salt stress. *Bacillus* and *Pseudarthrobacter* significantly increased in salt-treated spermosphere soil groups compared with CS25 and CS20 via taxonomic analysis and qPCR assays (Figure 3 and Figure 8). The two dominant bacteria genera in salt-treated spermosphere soil groups have previously been identified as common plant growth-promoting bacteria (PGPRs) [35,36,37,38]. Phosphorus is one of the essential mineral elements for plant growth, which is one of the major constituents in energy metabolism and biosynthesis of nucleic acids and cell membranes [39,40]. Most soil phosphorus is the organic form, which is poorly utilized by plants [39,41]. *Bacillus* can produce specific phosphatases to sequential mineralize organic phosphorus into inorganic phosphorus for plants [42]. In addition, *Bacillus* can also activate some signaling pathway to promote the growth of plants [43]. Thus, the higher abundance of *Bacillus* in salt-treated spermosphere soils may contribute to withstanding stress and promoting the emergence of seeds under salt stress. Interestingly, slightly higher phosphatase activity was detected within the salt-treated spermosphere compared with the untreated spermosphere in our study (Figure 7C), which may be associated with the increased numbers of *Bacillus* in these soils. The other salt-induced bacterium, *Pseudarthrobacter*, are also identified as beneficial bacteria, which can survive in the arid area or hostile environment containing high concentrations of heavy metals [44,45]. Overall, these results suggest that some seed-associated beneficial bacteria have positive effects on the peanuts, which may have implications for alleviating salt stress and enabling seed germination under salt stress conditions.

Soil enzymes are important bioactive proteins that participate in soil nutrient cycling and indicate soil fertility and soil environmental quality [28,46]. Urease is involved in soil nitrogen cycling and exerts an important role in improving soil quality and fertility [30,47]. Phosphatase plays an important role in hydrolyzing soil organic phosphate into inorganic phosphate, which can meet the phosphorus requirement of plants [31]. Invertase activity is related to soil carbon cycling, microbial biomass, and soil respiration intensity [30], and catalase activity is correlated with soil respiratory intensity and microbial activity, which can indicate the soil microbial life activities to some extent [29]. We found that invertase activity was lower in the presence of salt-treated spermosphere soils as compared to untreated peanut spermosphere soils (Figure 7), suggesting that the bacterial life activities are suppressed by soil salinity. However, higher phosphatase activity was detected in salt-treated spermosphere soils, which may be partly related to the increased number of the *Ballius* in these soils.

By studying the predicted function features of the bacterial community, we found that some metabolic functions (carbohydrate transport and metabolism, lipid metabolism, and metabolism of terpenoids and polyketides) were enriched in the salt-treated spermosphere soil groups (Figure 6). Carbohydrates such as trehalose and ectoine are known as important salt tolerance enhancers [48,49]. It has been well described that some secondary metabolites (terpenoids and polyketides) in PGPRs can also enhance salt tolerance and plant growth [50,51]. A previous study showed that some PGPRs can promote plant growth and salt stress tolerance via degrading xenobiotics and reducing pollutants of contaminated soils [52,53]. Thus, the more vigorous xenobiotics biodegradation and metabolism in salt-treated spermosphere soils may be implicated in salt stress tolerance (Figure 6B). Moreover, stress response processes (replication and repair and signaling molecules and interaction) predicted higher in salt-treated spermosphere soils, which can confer high tolerance levels to stress and toxic compounds [1]. Over the past decade, various studies have emerged that implicate members of the rhizosphere microbial communities in enhancing plants stress tolerance by activating stress response or providing a buffer zone for plants against stress [54,55,56]. In the previous paper, the defense mechanism in the microbial community of drought-treated peanut rhizosphere soils was significantly higher than that of the controlled soil [1]. Similar to this, the defense mechanism was also predicted as being higher in salt-treated peanut spermosphere soils in this study (Figure 6A). Together, the spermosphere bacterial communities may help peanuts survive abiotic stress by activating defense mechanisms. In a further study, we will propagate and inoculate moderately beneficial bacteria into the spermosphere soil and analyze the correlation between germination rate of various peanut cultivars and specific soil bacteria under salt stress conditions. Managing spermosphere microbes and delivering high populations of the desired microbes in pelletized seed coating are crucial to the effectiveness of cropping practices in the future.

## 4. Materials and Methods

### 4.1. Plant Materials and Seed Treatment

Peanuts Huayu20 (salt-sensitive peanut cultivar) and Huayu25 (salt-resistant peanut cultivar) were cultivated in the greenhouse at Laixi experimental station, China (120.53°E, 36.86°N), in 2018–2019 under the conditions of 28 °C and approximately 16/8 h light/dark photoperiod. In order to characterize the bacterial community in field conditions and further perform salt stress treatment, fine peanuts were grown in a transparent acrylic tank (7 cm in diameter and 8 cm tall) with tiny holes in the bottom. The topsoil was dug from a peanut field at Laixi experimental station and further sieved with a 1 cm sieve. Then the topsoil was dried in an oven until constant weight and 350 g soil were added to each tank. The physiochemical properties of soil were examined as follows: pH 6.7, EC 0.26 ds/m, organic matter 15.2 g/kg, total nitrogen 1.6 g/kg, available phosphorous 45.1 mg/kg, and available potassium 102.5 mg/kg. The salt-treated samples were added the required amount of NaCl in the soil and stirred well to blend to attain to 3.0 g/kg salt concentration before planting peanuts. After 12 h, two sterile peanuts of Huayu20 (salt-susceptible peanut cultivar) or Huayu25 (salt-resistant peanut cultivar) were planted to each transparent acrylic tank at a depth of 3 cm. Then, we placed them at the greenhouse under the conditions of 28 °C and approximately 16/8 h light/dark photoperiod. Subsequently, each transparent acrylic tank was irrigated with sterile ddH_2_O to keep the soil water content at 85% of field capacity by weighing, as was done in the previous study [1]. After 72 h (when most of the radicles can emerge), seeds and soil samples were collected and placed on the sterile silver paper [21], and the germination rate of the two peanut cultivars are shown in Figure S5. All the experiments were performed with three replicates per soil-treatment combination.

### 4.2. Samples of Spermosphere Compartments Collection and DNA Extraction

Spermosphere soil samples were composite samples containing seed surface soils and soils around the seeds. Briefly, the soils within a 10 mm radius around the seeds were sampled as soils around the seeds. For seed surface soils, germination seeds were placed in 20 mL of sterile centrifuge tube containing 10 mL PBS buffer (pH 7.0, per liter 0.0633 g of NaH_2_PO_4_∙H_2_O, 0.1650 g of Na_2_HPO_4_∙7H_2_O, 2 mL Silwet L-77). The bacterial community was separated by thorough centrifuging to remove the seed surface soil, and was then filtered through a 100 mm mesh cell strainer to remove seed debris and large soil aggregates. The filtrate was centrifuged again at 5000× *g* for 15 min and collected in a new 50 mL centrifuge tube. Then, 1 mL PBS buffer was added to the centrifuge tube to suspend the pellet, and the samples were then stored at −80 °C. All the experiments were performed in triplicate per soil-treatment combination. Spermosphere soil genomic DNA was extracted by MO BIO’s PowerSoil DNA Isolation Kit (Carlsbad, CA, USA) according to the manufacturer’s instructions.

### 4.3. 16S rRNA Gene Sequencing

Extractive DNA quality and concentrations were checked by 0.8% agarose gel electrophoresis and ultraviolet spectrophotometry. High-quality DNA samples were used for bacterial 16S rRNA gene amplification by Beijing Biomarker (Beijing, China). The specific primers 338F (forward primer, 5’- ACTCCTACGGGAGGCAGCA-3’) and 806R (reverse primer, 5’- GGACTACHVGGGTWTCTAAT-3’) with barcode were used for bacterial 16S rRNA tags (V3 and V4 regions) amplification. Bacterial DNA spermosphere samples were amplified with three phases of PCR program: a first phase consisting of 95 °C for 5 min, 30 cycles of 95 °C for 30 s, annealing at 50 °C for 30 s, and 72 °C for 60 s, and then a final extension at 72 °C for 7 min. PCR amplification was performed with 2× Phanta Max Master Mix (P515, Vazyme, Nanjing, China), and PCR products were checked by 0.8% agarose gel electrophoresis and further purified with Tiangen Gel Extraction Kit (Tiangen, Beijing, China). Sequencing libraries were produced by using TruSeq DNA PCR-Free Sample Preparation Kit (Illumina, San Diego, CA, USA) following the manufacturer’s instructions. The library was sequenced on an Illumina HiSeq 2500 platform and 250 bp paired-end reads were generated. Raw reads were submitted to Trace Archive National Coalition Building Institute (NCBI) Sequence Read Archive (SRA) with SRA accession SUB6484004 and bioproject accession PRJNA580321; the database is accessible via the following link: https://www.ncbi.nlm.nih.gov/sra/PRJNA580321.

### 4.4. Bioinformatics Analysis

The paired-end reads were merged and assembled into single sequences with PANDAseq [57]. Raw tags were preliminarily screened to obtain the high-quality clean tags according to the Trimmomatic software v0.33 quality-controlled process. Clean tags were compared with the reference Gold database (http://drive5.com/uchime/uchime_download.html) to discarded chimera sequences and effective tags finally obtained by using USEARCH (v5.2.236, http://www.drive5.com/usearch/) [54,58]. Then, the effective tags were classified as an operational taxonomic unit (OTU) with a 97% threshold [59]. We pick a representative sequence for each OTU and the taxonomy of each OTU representative sequence was assigned and annotated using the Ribosomal Database Project (RDP) classifier with a minimum bootstrap threshold of 80% [60]. The heat-map visualization with hierarchical clustering of the top 20 most abundant genera was generated using R-package, gplots (version 3.3.1), and color-coded by row z-scores [61].

### 4.5. Alpha and Beta Diversity Analysis

Perl scripts were used to analyze alpha (within samples) and beta (among samples) diversity of the spermosphere bacterial community. Rarefaction curves evaluate the species richness and sequence depth were made using QIIME [62]. The species accumulation curve was used to reflect whether the sequence depth is sufficient to estimate community richness [63]. The rank abundance curve reflecting the species abundance and evenness was performed using the specific bioinformatics software (http://en.wikipedia.org/wiki/Rank_abundance_curve) [64]. Each OTU representative sequence was used for taxonomic identification of each soil sample at five classification levels (phylum, class, order, family, and genus).

Beta diversity analysis was conducted to examine the similarity and dissimilarity of the community structure among different soil samples. The PCA analysis was performed on the community composition structure at the genus level to explore the similarities or dissimilarities among the various soil groups, which was applied to reduce the dimension of the original variables using the QIIME software package [65]. UPGMA analysis mainly refers to the hierarchical clustering analysis method using any distance to evaluate the similarity or dissimilarities among the soil groups [66]. Statistical testing among variation in bacterial community composition was carried out using ANOSIM analysis [67].

### 4.6. LEfSe and Metabolic Functional Prediction

LEfSe as an algorithm for high-dimensional biomarker was used for the quantitative analysis of biomarkers within different soil groups. This method can provide biological class explanations to establish statistical significance, biological consistency, and effect-size estimation of predicted biomarkers [68]. LEfSe analysis was performed on the website http://huttenhower.sph.harvard.edu/galaxy. The differential features were identified on the OTU level. LEfSe analysis was performed with the alpha value for the factorial Kruskal-Wallis test among classes was <0.05 and the threshold on the logarithmic LDA score for discriminative features was >3.5.

Metabolic functional features of the bacterial community via 16S rRNA gene amplification-based high-throughput sequencing data were predicted using the phylogenetic investigation of communities by reconstruction of unobserved states (PICRUSt) and Kyoto Encyclopedia of Genes and Genomes (KEGG) pathways according to the previous study [69].

### 4.7. Quantification of Predominant Phyla or Genera in the Peanut Spermosphere Samples

qPCR was used to analyze the main bacterial phyla (Proteobacteria, Firmicutes, Actinobacteria, and Bacteroidetes) and beneficial bacteria (*Bacillus*) following the methods described in the previous study [70]. qPCR analysis was performed by ChamQ SYBR Color qPCR Master Mix (Q411, Vazyme, Nanjing, China), and Bio-Rad CFX96 (Bio-Rad, Hercules, CA, USA) with the following reaction mixture: 0.5 µL of primers (10 µM), 7.5 µL ChamQ SYBR Color qPCR Master Mix, and template DNA (20 ng of total soil DNA or plasmid DNA for standard curves). The primers are listed in Table S4.

### 4.8. Soil Enzyme Assays

The potential activities of several soil enzymes were quantified according to the previous study [29]. Invertase activity: 2 g of fresh spermosphere soil was added into a volumetric flask containing 15 mL of 8% sucrose, 5 mL of phosphate buffer (pH 5.5), and 5 drops of methylbenzene, and then the volumetric flask was placed in 37 °C incubators for 24 h. Subsequently, we filtrated the solution and 1 mL of filtrate was incubated with 3 mL of 3,5-dinitrosalicylic acid (DNS) in a boiling bath for 5 min. Finally, the solution was diluted to 100 mL, and absorbance at 508 nm was measured using a spectrophotometer; urease activity: 5 g of fresh spermosphere soil was incubated with 1 mL of methylbenzene for 15 min. Then, 10 mL of 10% urea and 20 mL of citrate buffer (pH 6.7) were incubated with the spermosphere soil at 37 °C for 24 h. After filtration, 3 mL of filtrate was mixed with 4 mL of sodium phenoxide and 3 mL of sodium hypochlorite. Finally, the solution was diluted to 50 mL, the absorbance at 578 nm was measured using a spectrophotometer; catalase activity: 2 g of fresh spermosphere soil was mixed with 40 mL of distilled water and 5 mL of 0.3% H_2_O_2_ in a flask. Then, the flask was sealed and shaken at 120 rpm for 20 min. A total of 5 mL of 1.5 M H_2_SO_4_ was added to the flask to terminate the reaction. Finally, the solution was filtrated, and 25 mL of filtrate was titrated with KMnO_4_; phosphatase activity: soil neutral phosphatase activity (soil pH 6.7) was examined by using Solarbio Soil Neutral Phosphatase (S-NP) Activity Assay Kit (BC0465) and the absorbance at 660 nm was measured using a spectrophotometer.

### 4.9. Data Availability

The datasets generated for this study can be found in Trace Archive NCBI Sequence Read Archive (SRA) with SRA accession SUB6484004 and bioproject accession PRJNA580321; the database is accessible via the following link: https://www.ncbi.nlm.nih.gov/sra/PRJNA580321.

### 4.10. Statistical Tests

All statistical analyses were conducted with the R program (v3.3.0, https://www.r-project.org/), and 999 displacement tests were performed to determine whether the differences between the salt-treated and untreated soil groups were statistically significant.

## Supplementary Materials

Supplementary materials can be found at https://www.mdpi.com/1422-0067/21/6/2131/s1.

## Abbreviations

Huayu25	salt-resistant peanut cultivar
SS25	salt-treated spermosphere around Huayu25
LDA	linear discriminant analysis
LEfSe	LDA effect size
KEGG	Kyoto Encyclopedia of Genes and Genomes
qRT-PCR	quantitative real-time polymerase chain reaction
