# Supplementary material for: Influence of Salt Stress on Growth of Spermosphere Bacterial Communities in Different Peanut (Arachis hypogaea L.) Cultivars
Source: Int J Mol Sci. 2020 Mar 20;21(6):2131. doi: 10.3390/ijms21062131 (PMC7139419; doi:10.3390/ijms21062131)
Supplement: Supplementary file 1 [file ijms-21-02131-s001.pdf]

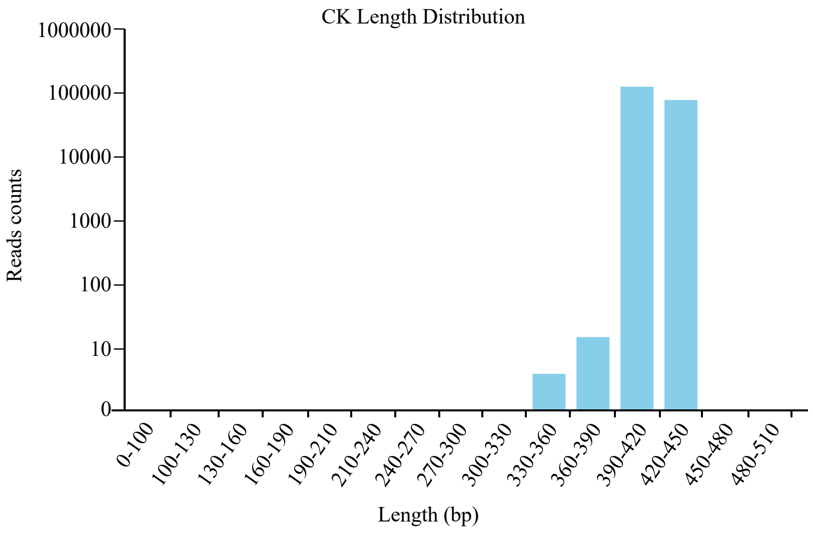

Supplementary Figure S1. Length distribution of trimmed sequences in peanut spermosphere soils and bulk soils.

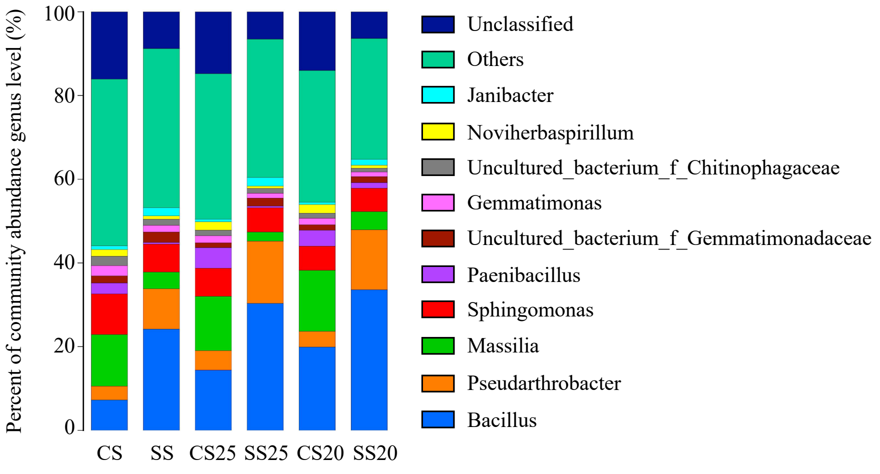

Supplementary Figure S2. Bacterial community structure in peanut spermosphere soils and bulk soils at the genus level. The relative abundance is calculated by averaging the abundances of three duplicates in each soil group.

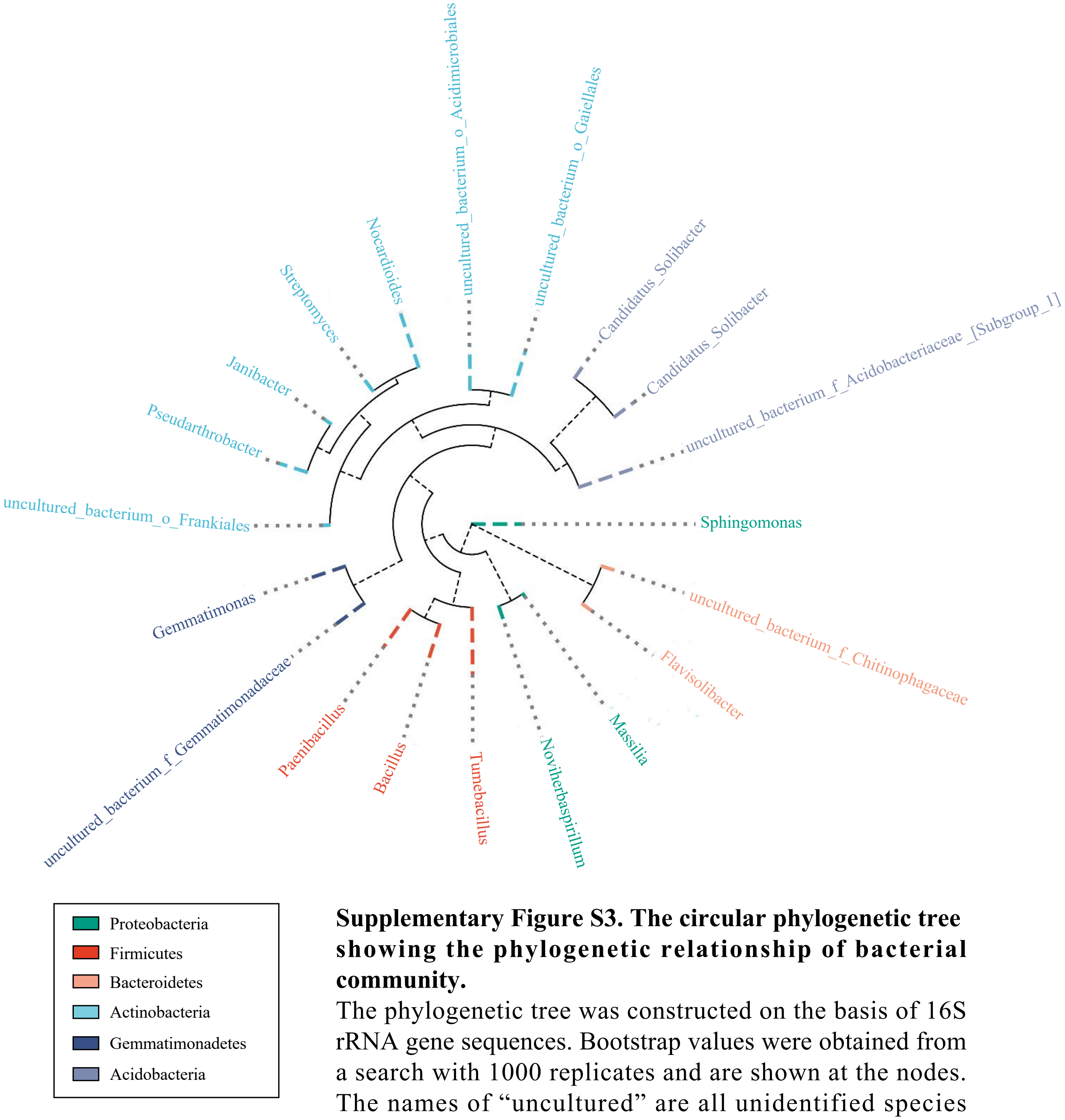

Supplementary Figure S3. The circular phylogenetic tree showing the phylogenetic relationship of bacterial community.
The phylogenetic tree was constructed on the basis of 16S rRNA gene sequences. Bootstrap values were obtained from a search with 1000 replicates and are shown at the nodes. The names of "uncultured" are all unidentified species obtained directly from database via sequence alignment.

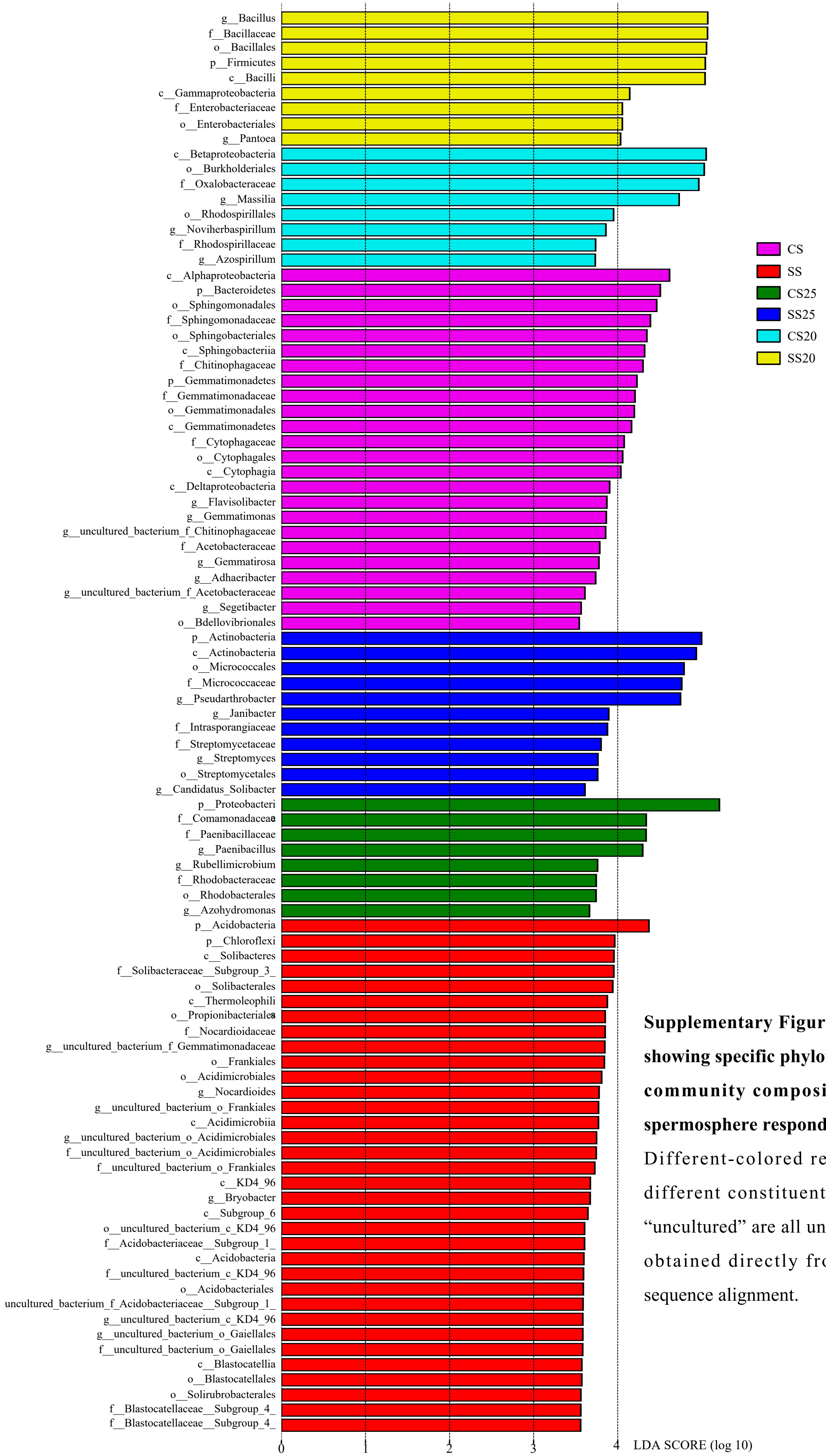

Supplementary Figure S4. Cladogram showing specific phylotypes of bacterial community compositions of peanut spermosphere responding to salt stress. Different-colored regions represent different constituents. The names of "uncultured" are all unidentified species obtained directly from database via sequence alignment.

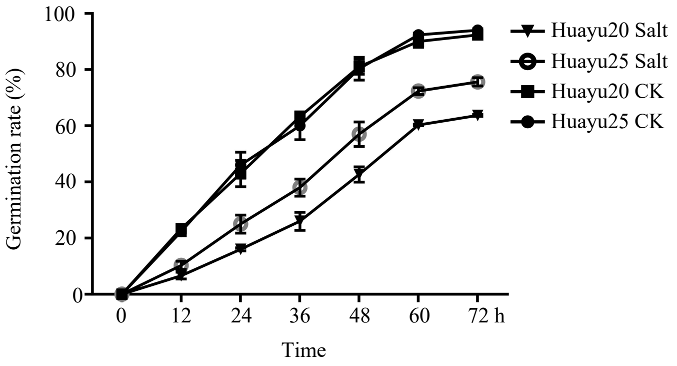

Supplementary Figure S5. The germination rates of two peanut cultivars under salt stress and normal conditions. Seeds (Huayu20 and Huayu25) germinated for 72 h under salt stress and normal conditions. Germination rates were measured every 12 h.

Supplementary Table S1. Distribution and abundance of taxa at the genus level.

genus	CS	SS	CS25	SS25	CS20	SS20
Bacillus	0.072514	0.242047	0.143987	0.303410	0.199150	0.336021
Pseudarthrobacter	0.032876	0.096123	0.046488	0.148391	0.037420	0.142998
Massilia	0.123413	0.039911	0.129560	0.022057	0.145766	0.043473
Sphingomonas	0.097053	0.066825	0.067238	0.057823	0.057680	0.056146
Paenibacillus	0.026084	0.003814	0.049206	0.004088	0.038018	0.013148
Uncultured_bacterium_f_Gemmatimonadaceae	0.017124	0.025220	0.011519	0.019109	0.013137	0.014404
Gemmatimonas	0.024475	0.015743	0.016904	0.011425	0.015447	0.011095
Uncultured_bacterium_f_Chitinophagaceae	0.022446	0.014703	0.013455	0.011877	0.012214	0.009135
Noviherbaspirillum	0.015884	0.007989	0.019798	0.005467	0.020707	0.006885
Janibacter	0.008666	0.019453	0.006324	0.020477	0.005509	0.014131
Others	0.398646	0.380252	0.347578	0.330415	0.314573	0.288672
Unclassified	0.160820	0.087921	0.147944	0.065462	0.140379	0.063893

Supplementary Table S2. The relative abundance and diversity of functional groups in various peanut spermosphere soil groups and bulk soil groups in the context of the Cluster of Orthologous Groups (COG) database.

Class	CS	SS	CS25	SS25	CS20	SS20
Amino acid transport and metabolism	0.081448	0.081396	0.083173	0.082306	0.083479	0.083797
Carbohydrate transport and metabolism	0.062661	0.067563	0.062775	0.070310	0.063005	0.070992
Cell cycle control, cell division, chromosome partitioning	0.008600	0.008623	0.008737	0.008778	0.008789	0.008928
Cell motility	0.022625	0.017201	0.024087	0.015507	0.024787	0.017415
Cell wall/membrane/envelope biogenesis	0.062366	0.059961	0.060307	0.058143	0.060217	0.057395
Chromatin structure and dynamics	0.000541	0.000440	0.000558	0.000410	0.000580	0.000417
Coenzyme transport and metabolism	0.040966	0.041611	0.040531	0.041729	0.040253	0.041133
Cytoskeleton	0.000129	0.000124	0.000130	0.000108	0.000140	0.000094
Defense mechanisms	0.017166	0.019080	0.016199	0.018918	0.016179	0.017825
Energy production and conversion	0.059813	0.061060	0.058698	0.060897	0.057850	0.059300
Extracellular structures	0.000021	0.000097	0.000028	0.000007	0.000030	0.000011
Function unknown	0.081295	0.079118	0.083009	0.079607	0.083402	0.081696
General function prediction only	0.119595	0.123991	0.118385	0.124788	0.118185	0.123145
Inorganic ion transport and metabolism	0.048667	0.046687	0.049379	0.046910	0.048986	0.048362
Intracellular trafficking, secretion, and vesicular transport	0.027797	0.023860	0.027828	0.022421	0.028050	0.023099
Lipid transport and metabolism	0.041318	0.041981	0.040807	0.042025	0.040179	0.040827
Nuclear structure	0.000000	0.000000	0.000000	0.000000	0.000000	0.000000
Nucleotide transport and metabolism	0.020091	0.021311	0.020267	0.021842	0.020249	0.021793
Posttranslational modification, protein turnover, chaperones	0.036271	0.034835	0.036069	0.034382	0.036020	0.034326
RNA processing and modification	0.000417	0.000403	0.000438	0.000411	0.000431	0.000406
Replication, recombination and repair	0.048297	0.051484	0.047437	0.052710	0.047399	0.051460
Secondary metabolites biosynthesis, transport and catabolism	0.028313	0.028585	0.028051	0.028563	0.027713	0.027923
Signal transduction mechanisms	0.067098	0.063187	0.067674	0.060800	0.068374	0.061166
Transcription	0.078200	0.080694	0.079509	0.081449	0.080126	0.081814
Translation, ribosomal structure and biogenesis	0.046303	0.046795	0.045926	0.046978	0.045577	0.046674

Supplementary Table S3. The relative abundance and diversity of functional groups in various peanut spermosphere soil groups and bulk soil groups in the Kyoto Encyclopedia of Genes and Genomes (KEGG) database.

Class	CS	SS	CS25	SS25	CS20	SS20
Amino acid metabolism	0.131018	0.134316	0.130865	0.135916	0.130607	0.134894
Biosynthesis of other secondary metabolites	0.012698	0.013786	0.012026	0.013945	0.011876	0.013283
Cancers: Overview	0.008563	0.008041	0.008792	0.008125	0.008832	0.008458
Cancers: Specific types	0.001069	0.000933	0.001054	0.000909	0.001022	0.000934
Carbohydrate metabolism	0.141747	0.149187	0.140262	0.151840	0.140418	0.150641
Cardiovascular diseases	0.000252	0.000247	0.000213	0.000235	0.000213	0.000209
Cell growth and death	0.007346	0.007040	0.007140	0.006884	0.007028	0.006779
Cell motility	0.022298	0.017042	0.024065	0.015518	0.024897	0.017689
Cellular community	4.30E-06	5.90E-06	3.57E-06	5.39E-06	1.30E-05	3.57E-06
Circulatory system	0.000651	0.000434	0.000670	0.000371	0.000658	0.000396
Development	0.000000	0.000000	0.000000	0.000000	0.000000	0.000000
Digestive system	0.000766	0.000604	0.000806	0.000573	0.000863	0.000669
Drug resistance	0.006841	0.006134	0.006755	0.005821	0.006845	0.005950
Endocrine and metabolic diseases	0.001951	0.001680	0.001952	0.001604	0.001943	0.001622
Endocrine system	0.007185	0.007300	0.007053	0.007129	0.007079	0.006821
Energy metabolism	0.066677	0.067466	0.065830	0.066988	0.065236	0.065683
Environmental adaptation	0.001697	0.001518	0.001682	0.001455	0.001682	0.001508
Excretory system	0.000651	0.000647	0.000655	0.000635	0.000669	0.000628
Folding, sorting and degradation	0.017457	0.017993	0.017266	0.018091	0.017201	0.017932
Global and overview maps	0.126735	0.129461	0.125595	0.130079	0.124918	0.128582
Glycan biosynthesis and metabolism	0.015875	0.015875	0.014859	0.015194	0.014934	0.014648
Immune diseases	0.000766	0.000823	0.000775	0.000851	0.000776	0.000830
Immune system	0.000521	0.000436	0.000570	0.000430	0.000582	0.000491
Infectious diseases: Bacterial	0.007613	0.006956	0.007734	0.006740	0.007869	0.007059
Infectious diseases: Parasitic	0.001093	0.001048	0.00104	0.001029	0.001065	0.001008
Infectious diseases: Viral	0.000587	0.000521	0.000516	0.000503	0.000470	0.000473
Lipid metabolism	0.044074	0.045023	0.043984	0.044912	0.043912	0.044364
Membrane transport	0.047064	0.040737	0.050432	0.039929	0.051796	0.044221
Metabolism of cofactors and vitamins	0.061210	0.060996	0.060847	0.060776	0.060740	0.060680
Metabolism of other amino acids	0.028801	0.027673	0.029141	0.027554	0.029266	0.028006
Metabolism of terpenoids and polyketides	0.024795	0.026074	0.024479	0.026026	0.024130	0.025122
Nervous system	0.002683	0.002850	0.002582	0.002795	0.002605	0.002651
Neurodegenerative diseases	0.004532	0.003847	0.004564	0.003712	0.004473	0.003860
Nucleotide metabolism	0.041551	0.041715	0.041528	0.041853	0.041561	0.042003
Replication and repair	0.031030	0.032240	0.030448	0.032583	0.030348	0.032206
Sensory system	1.42E-06	1.91E-06	1.11E-06	1.64E-06	4.31E-06	1.16E-06
Signal transduction	0.040762	0.035800	0.042772	0.034614	0.043805	0.037071
Signaling molecules and interaction	1.07E-06	3.41E-06	2.73E-06	4.34E-06	2.25E-06	3.85E-06
Substance dependence	0.000816	0.000857	0.000695	0.000777	0.000740	0.000682
Transcription	0.001704	0.001799	0.001709	0.001828	0.001705	0.001800
Translation	0.034977	0.035797	0.034265	0.035671	0.033985	0.034976
Transport and catabolism	0.004789	0.004897	0.004626	0.004796	0.004594	0.004582
Xenobiotics biodegradation and metabolism	0.049149	0.050197	0.049748	0.051296	0.048637	0.050580

Supplementary Table S4. Primers used in this study.

Purpose	Name	Sequence	Reference
qPCR	Alphaproteobacteria F	ACTCCTACGGGAGGCAGCAG	Noah Fierer *et al*. Appl. Environ. Microbiol. 2005, 71, 4117–4120
	Alphaproteobacteria R	TCTACGRATTTCACCYCTAC	
	Betaproteobacteria F	ACTCCTACGGGAGGCAGCAG	Noah Fierer *et al*. Appl. Environ. Microbiol. 2005, 71, 4117–4120
	Betaproteobacteria R	TCACTGCTACACGYG	
	Actinobacteria F	CGCGGCCTATCAGCTTGTTG	Noah Fierer et al. Appl. Environ. Microbiol. 2005, 71, 4117–4120
	Actinobacteria R	ATTACCGCGGCTGCTGG	
	Firmicutes F	GCAGTAGGGAATCTTCCG	Noah Fierer et al. Appl. Environ. Microbiol. 2005, 71, 4117–4120
	Firmicutes R	ATTACCGCGGCTGCTGG	
	Acidobacteria F	GTYAACTCGGAGGAAGGT	Noah Fierer *et al*. Appl. Environ. Microbiol. 2005, 71, 4117–4120
	Acidobacteria R	CTGATCTGCGATTACTAGCGACTCC	
	Bacillus F	GGGAAACCGGGGCTAATACCGGAT	Noah Fierer et al. Appl. Environ. Microbiol. 2005, 71, 4117–4120
	Bacillus R	CGGTGTGTACAAGGCCCGGGAACG	
